# Theorising health equity research for people with intersex variance through new materialism

**DOI:** 10.1111/1467-9566.13561

**Published:** 2022-10-10

**Authors:** Laetitia Zeeman, Kay Aranda

**Affiliations:** ^1^ School of Sport and Health Sciences University of Brighton Brighton UK; ^2^ Centre for Transforming Sexuality and Gender University of Brighton Brighton UK

**Keywords:** assemblage, equity, feminism, gender, health, inequalities, intersex, new materialism, sex, variance

## Abstract

Health inequalities impact sex‐variant people in highly differentiated ways. This is evidenced in much academic and activist intersex research documenting the highly specific forms of inequalities arising from misrecognition, discrimination and human rights abuses inherent to pathologised accounts of non‐normative bodies. Important theoretical work further interrogates the implications of sex variant subjectivities, identities and bodies for static or binary notions of both sex and gender. In this paper, we aim to contribute further to this scholarship. We draw upon feminist materialist and Deleuzean‐informed understandings of materials or matter to rethink debates over sex‐variant subjectivities, identities and bodies in relation to inequalities in health. We argue ‘the turn to matter’ and associated new materialist theories draw attention to the complex, dynamic relational assemblages and entanglements mutually constituting the affective, embodied and socio‐material worlds of intersex people. Informed by these theories, we propose that inequalities can be more fully addressed through a new health equity research agenda that is co‐produced with sex‐variant people. This agenda will enable a fuller exploration of the unsettling but transformative capacities of intersex matters and meanings with the contextually specific understandings of equity in relation to health and health care.

## INTRODUCTION

It is not enough to argue that there is no prediscursive “sex” that acts as the stable point of reference on which, or in relation to which, the cultural construction of gender proceeds. To claim that sex is already gendered, already constructed, is not yet to explain in which way the “materiality” of sex is forcibly produced. What are the constraints by which bodies are materialised as “sexed,” and how are we to understand the “matter” of sex, and of bodies more generally…? Which bodies come to matter—and why?

(Butler, 1993, p. xi)

Intersex variance is often represented as people being born with, or later in life, developing sex variation as physical, hormonal or genetic features relating to bodily markers (Köhler et al., 2012). Intersex‐variant bodies in both academic and activist research are argued to further question or trouble the notion of static, binary understandings of sex that become categorised reductively as either male or female. Rather, intersex embodiment exceeds normative notions of fixed male and female binary sex (Zeeman & Aranda, 2020). Much Western activist and academic research seeking to give voice and documenting the misrecognition of those living with sex variance also reveals how health care provision in developed welfare systems is inherently pathologising and oppressively normative. This normativity is systemic as well as interpersonal, resulting in encounters that are frequently stressful or discriminatory for people with sex‐variant, non‐normative bodies and lives (Hassan et al., 2018; Zeeman & Aranda, 2020; Zeeman et al., 2014). Meer and Muller (2017) further argue that in these health‐related spaces, accessing health and care actively constitute the identities of people in two specific ways: firstly materially—in terms of their health, and secondly affectively—through levels of wellbeing and identity formation. This is key to our argument and our exploration in this paper, which is to ‘understand the matter of sex’ (Butler, 1993, p. xi), and the potential of sociomaterial theories or new materialisms to rethink understandings of health, wellbeing and health equity for those living with intersex variance. We understand health equity to refer to the absence of unfair, avoidable or amendable differences among people who experience social‐ and health‐related disadvantages and inequalities. So, though related to inequalities, health equity is distinct in aims and focus. It is achieved when all can reach their full potential for health and wellbeing. Hence, the focus is on resources and policies for those groups who experience exclusion and discrimination (based on sex, gender, race, age, disability etc). By means of concerted efforts, marginal groups can attain improved health and living conditions faster than those who are in a better position (WHO, 2021).

Whilst we fully acknowledge the harms and inequalities documented to date, we wish to more positively consider what intersex bodies can be, do and know in relation to health, wellbeing and health equity. We argue that the aforementioned theories have the potential to inform understandings of how intersex variant people experience normativity; this is both in how lives are lived, but equally how such normativity and non‐normativity is strategically utilised, subverted or displaced, resisted or replaced or even desired (Zeiler, 2020). These theories have the potential to account for the dynamic complexity of people’s lives and allow for different answers to the following question: *What does health equity and embodied wellbeing look like or feel like for people with intersex variance?*

Next, we discuss the health inequalities faced by people with intersex variance before going on to outline the potential of new materialist thinking. We then explore more specifically health‐related empirical examples to illustrate how these theories deepen or change our understandings. Finally, we discuss how these frames of understanding shape research methodologies and how a co‐produced agenda would proceed and be conducted.

### Turning to matter

Emerging empirical research documenting health inequalities for people with intersex variance is increasing and is often associated with their experiences or encounters with health care. Much of this work shows the significance of temporality to sex‐variant people, who encounter medicine across their lifespan, being subjected to medical scrutiny and interventions at a young age or later in life (Jones, 2018). For some, these interventions may occur without informed consent or without their knowledge (Köhler et al., 2012). Research shows where there is limited understanding of sex variation, people may experience discrimination, social isolation and stigma, leading to higher rates of psychological distress, anxiety and depression compared to the general population (Jones, 2016). More specifically, empirical intersex variance research documents how this biomedical scrutiny can manifest longer term later in life as depression, anxiety, even hypertension and cognitive difficulties (Rosenwohl‐Mack et al., 2020). This means that for many people with intersex variance, though not for all, interactions with medical systems, processes and practices may be confusing or stressful and problematic (Meoded Danon, 2019; Thyen et al., 2014). For example, some people with intersex variance report dissatisfaction with their treatment and surgery to feminise or masculinise their bodies, while others decline any invasive medical interventions entirely (Thyen et al., 2014; Zeeman & Aranda, 2020). Conversely, parents are sometimes advised to delay decision‐making about raising their child in a specific gender as related to sex until the young person can self‐identify (Köhler et al., 2012; Schweizer et al., 2014). Progressive health care treatments such as these are seeking to question normative binaries of sex and gender and promote more diverse understandings of sex variance and non‐binary gender (Schweizer et al., 2014). Central to our argument in this paper though is how to move these debates beyond further documenting the harms or indeed benefits for some, or moral arguments for more inclusive social recognition of minority difference. We argue that the use of new materialist theories has the potential to shift these foci to reveal the complex nature of sex‐variant people’s capacities and potentialities more positively for wellbeing, and in turn how to promote health equity.

Our starting point is the recent ‘turn to matter’, increasingly evident in health and social research. This suggests that new materialist theories have value for revised understandings of the body, sexual difference, agency, power and social change (Alldred & Fox, 2017; Andrews & Duff, 2019; Aranda, 2019; Fox & Powell, 2021; Fullagar, 2017; Jagger, 2015; Lupton, 2019; Markula, 2019). However, the full implications of these sociomaterialist theories over questions of inequalities, difference, and power, as well as in empirical health research, are yet to be realised (Alaimo & Hekman, 2008; Braidotti, 2019; Coole & Frost, 2010; Fox & Powell, 2021). Materialism has always been present in theorising health and health care. This is evident in earlier waves of feminism that rejected material explanations in order to avoid essentialist or biologically reductionist readings of the female body. Modern feminisms preferred instead a focus on the sociality of gender with its implied potential for change. These conceptions of matter were, however, often instrumental, often viewing materials for use value or as determining, fixed or static and inert. In relation to health inequalities, for example, this is evident in accounts of the social determinants of inequality, such as income, employment, living conditions and education (Marmot & Allen, 2014). The damaging effects of social inequalities that manifest in socioeconomic deprivation or disadvantage are well known to lead to significant, seemingly entrenched and increasing physical, mental and subjective ill health, including increased shame and loss of self‐esteem or status (Piketty, 2014; Whitehead, 1991; Wilkinson & Pickett, 2018). Given these material disadvantages and inequalities in health are acknowledged to be preventable and therefore evidently unfair and unjust, broader moral questions are raised over societal responsibilities for fairness and justice, with demands for policies and resources to address these injustices.

However, critiques of inequality research suggest that there may be more fundamental problems than a lack of political will or resources. The limits of these accounts and responses can be found in the underlying, often latent, humanist and emancipatory modern political philosophy. Here, notions of individuals and collectivities act upon the material world to address inequalities or find solutions through exposing hidden oppressive forces or structures or discourses to bring about progressive change (Aranda, 2019; Fox, 2016). Yet it is clearly evident that such assumptions have failed to transform inequality, and justice remains elusive. The recent revised interest in understanding materialism or materiality in health and social theory does not do away with but continues an ongoing critique of humanism or humancentric views of the world. Feminist materialism, including post‐human feminism and the Deleuzean concept of assemblages (Deleuze, 1988; Fox & Powell, 2021), extends our understanding and thinking of inequalities and potentially equity in health. With emergent understandings of the world as always materialising, the notion of matter combined with discursive meaning as inter‐ and even intra‐related, or as indivisible, suggests that we need radical revisions of everyday, modern or humanist conceptions of agency, as well as power and change (Alaimo & Hekman, 2008; Braidotti, 2013, 2019). The focus on meaning and matter as always intimately connected (Barad, 2007) erases the separation between matter as the physical world and meaning as the sociolinguistic world.

New materialism therefore offers a new lens and opportunity to reconsider material entities such as health care places, settings (the clinic), or bodies, and practices (surgery/wellbeing/health), and how these combine with meanings. These meanings and matters rely on specific assumptions and concepts. For example, the work of Judith Butler drew attention to the material nature of the body, including sex, but without reinstating the materiality of the body as foundational (Barad, 1998). One of the tools utilised to disrupt this foundational materiality is Butler’s theory of performativity (Butler, 2004). Performativity proposes instead an undecided or processual nature of what was once considered fixed, static or settled. Karen Barad questions whether Butler’s notion of materialisation goes far enough to displace the active versus passive duality of matter (Barad, 1998, p. 91). Although Butler sets out to critique matter as a fixed, bounded entity via the discursive construction of matter, she fails to suggest ‘how matter comes to matter’ (Barad, 1998, p. 91). This is a key starting point for Barad and most new materialist theories, which claim to speak to the relationship between the discursive and the material. However, Barad does see Butler’s account of materiality as significant as it is where matter plays an active and interactive part in the world’s becoming (Barad, 1998, 2003). A further important concept in these debates is the Deleuzean concept of ‘affect’ as a capacity of matter that involves both the ability to ‘affect and be affected’ (Deleuze, 1988, pp. 123–126; Fox & Powell, 2021). This capacity extends to all matter, whether human or non‐human, reflecting the dynamism of matter, rather than seeing matter as set or stagnant. The ability of matter to ‘affect and be affected’ represents the relational ability or the relational ontology of ‘affect’, where non‐human matter (such as a clinical setting and the physical surroundings) can affect the body and, similarly, the body can affect the physical setting. The ability to ‘affect and be affected’ leads to the third concept of ‘capacity’, where new materialists perceive the material world, including all matter as changeable and evolving. As such, all matter is considered lively, with capacities to affect. The focus for research is then on actions, events and different interactions, which are affects that form constellations or assemble in particular ways. Assemblages are a further key analytical resource. This is a view of the world as constantly developing through networks of habitual, spontaneous and non‐linear modes or ways of thinking and working. Composed of material or physical processes, assemblages include people or bodies, contexts, memories, histories, cultures, experiences, codes and normativities particular to an event (Alldred & Fox, 2017; Fox, 2016, 2017; Fox & Alldred, 2013). Bodies are a good example of these capacities, affects and assemblages, which we discuss next.

### Bodies materialising

For Deleuze (1988, p. 123), matter, including human bodies, have no ontological standing except for what is developed in assemblages. This is often bodies, with further transitory objects and concepts. Consequently, according to Fox and Powell (2021, p. 4), we do not ask what bodies, objects and concepts are, in terms of the nature of their reality, but what they can do (Buchanan, 1997), or what are their transformative abilities? What can they become? These capacities for transformation include both limitations and constraining factors, but as importantly, facilitative factors to create further options or change. These understandings have implications for rethinking materiality relevant to people with intersex variance. Complementing Deleuzean understandings of bodies, new materialist thinking similarly assumes that materiality is never a fixed inherent property of independently existing objects, things or bodies that are separate from humans. Thus, materiality for people with intersex variance is conceived as always a coming into being, with meaning, through language or discourse. With the body envisioned as vital energy at work in the world, the possibilities for change become more extensive than just the common focus on human intention and practices alone. Barad’s notion of ‘ontoepistemology’, for example, argues for this inseparability and mutual entanglement of ontology and epistemology rather than the separation or opposition of subject and object, or being and knowing. For Barad, the material and discursive have no ontological or epistemological prior existence. They cannot be explained in terms of the other, neither are they reducible to the other but instead, ‘matter and meaning are mutually articulated’ (Barad, 2007, p. 152).

Thus embodied experiences, materials, bodies, identities, culture and the social world; all are constituted through ‘matters of practices, doings and actions’ (Barad, 2007, p. 135). Her concept of *intra‐action* reinforces this permeability of boundaries and contests humanist binaries of object and subject or the concerns with the interaction between two separate bodies or entities. When these concepts are applied to understand the materiality of people with intersex variance, intra‐action, as opposed to interaction, better captures the entanglement of relations involved and the dynamic nature of the material. Although the specificity of any intra‐actions will always be shaped by specific configurations of power enacting this agency (Barad, 2012).

### Rethinking health as relational entanglements with the material and social worlds

In drawing on the work of key theorists such as Barad (2007), Braidotti (2013), Haraway (2008), Fox and Alldred (2016), together with Fox and Powell (2021), implies the blurring of non‐permeable spaces that are assumed to be bounded between species and environments. Together, these new materialist propositions suggest that we need to fully understand the entangled ontologies that blur the assumed divides between human and non‐human worlds or that suggest a more‐than‐human world (Andrews & Duff, 2019). As such, all that is in the world are the results of interactions between living and material things. These practices include the energies or flows and intensities produced as both human and non‐human actors interact (Andrews & Duff, 2019). For example, new materialist thinking moves on from the traditional materialist emphasis on the body as biological or social, affected by socioeconomic determinants or understandings of its social construction or lived experience as in the phenomenological body. Instead, new materialism suggests a reformulation of subjectivity and the centrality of humans as the foci for understandings and research. The human body, including sex variant embodiment, is understood to develop capabilities that are context‐specific. These capabilities or capacities benefit or limit our social position and opportunities, and hence the experience of inequalities over a lifespan. The social position of individuals is therefore never constant or singular, but instead consists of numerous moments of benefits and drawbacks generated by the interchange via the ability to affect and be affected by both people (human) and non‐human matter (Fox & Powell, 2021). These alternative accounts of bodies also suggest differing understandings of power and change (Aranda, 2019; Andrews & Duff, 2019; Fox & Alldred, 2016). A new materialist understanding of health inequalities starts with the body as an expanding ability to make new relations and generate alternative options for action and engagement (Fox & Powell, 2021). Within this framing, health becomes a capability to develop different relations, including connections between humans, but also connections between humans and non‐human matter. Such relations are mutually constitutive. Both health and illness and material disadvantage hold the capacity to increase or decrease the ability of the body as a vehicle for exchange with both the material and non‐material worlds. As Fox and Powell (2021, p. 4) suggest, ‘sociomaterial advantage/disadvantage may, respectively, establish or constrain the capacity for physical or mental wellbeing’ through the body. This is not new. What is different are the detailed ways in which this occurs as revealed by these theories, how and in what ways these advantages/disadvantages emerge. How power is conceived in relation to this or in how these are sustained, and how advantages/disadvantages could be changed.

We are arguing that new materialism has the potential to address the health of those living with intersex variance as relational entanglements or assemblages through which we can more deeply and fully understand the material realities of health, the constraints and possibilities, as well as the access or utilisation of health care. In terms of the sociology of health and illness, realist and constructionist accounts of the body are relatively well known (see Bendelow, 2009, e.g.). These explanations were often found to be limiting for several reasons. The realist body often works with an unexamined or unquestioned essentialism, which often leads to a hierarchical or over‐simplified homogenised set of categories of identity. This was helpful in drawing attention to marginalised or excluded groups in the first instance, what Butler (1993) argued to be a strategic essentialism when a collective voice is needed to further political goals (such as LGBTQI+ pride). Constructionist approaches, as Fox and Bale (2017) suggest, interrogate these limits and expose these flaws. However, such accounts offer rich critiques but suggest very little in terms of tackling entrenched discrimination in ‘real’ life nor how to sustain political and social change to promote greater health equity. The concept of assemblages could address these limitations and critiques.

### Examples of bodies‐health‐equity assemblages

As previously discussed, assemblages offer a view of the world as developing through networks composed of materials, contexts, memories, histories, bodies, cultures, experiences, and normativities particular to an event (Alldred & Fox, 2017; Fox, 2016, 2017; Fox & Alldred, 2013). For example, the materials of a health‐care assemblages include evolving biotechnology in health settings such as surgery, procedures, processes of diagnosis, systems of surveillance and other practices employed to maintain health (Andrews & Duff, 2019).

Fox and Bale (2017) provide helpful examples of how new materialism and assemblages can be utilised to analyse health and wellbeing in their research on the sexualisation of young people. In exploring the assemblages of bodies, things, ideas and social institutions involved, they show how relations and affects both produce the potential but also perpetuate limits in terms of discourse or common sense understandings of sexual activity for young people. They show how misogynistic sexual objectification and the commodification of bodies dominate concerns over sexuality and young people. This lens is used to focus not only on the meanings from interviews from participants but also the materialities of the phenomena—in this case sexualisation. This emphasis brings to the fore the interactions between bodies and matter, as in physical things, social institutions, as well as ideas, feelings, desires, memories and histories within a sexuality‐assemblage (Fox & Bale, 2017).

Further examples can be found in arguments for the assemblages of materials with bodies and individuals, and affect in studies of young people, disability and rehabilitation. Using new materialist theories, technology is conceived as part of health‐related assemblages of bodies, things and spaces that have capacities to enable or constrain. Gibson et al. (2017) show how these configurations materialise for young people living with disabilities. Using new materialist theories, Gibson et al. (2017) show how this thinking decentres the autonomous subject of western neoliberal health care to bring to the fore the interactions between human and non‐human worlds. In their studies of young people living with disabilities, they show how such theories create new spaces to interrogate people's abilities/inabilities and how these materialise or become configured in particular ways (Gibson et al., 2017). In contrast to biomedical accounts of function and ability, these theories offer fine‐grained analysis but not of individuals, but of socio‐technical interactions or practice. In so doing, they point to different, new spaces or places for positive interventions and progressive opportunities for change (Gibson et al., 2017). Similarly, public health and health geography scholars are exploring how the materiality of environments or technologies may transform our understandings of health inequalities and health subjectivities including embodiment (Andrews & Duff, 2019; Maller & Stengers, 2019).

Further examples can be found in research related to digital health technologies. As Fox (2017) and Lupton (2019) argue, conceptually, assemblages aim to avoid the separation of technology as a solution from its consideration as a social cultural phenomenon. Rather, the focus is on the complex relations that exist between both, but also the attachments to and rejection of materials and meanings, and how these flux and wane over time and place. When conceived as assemblages of human and non‐human relations, both authors argue different questions can be asked. Fox and Alldred (2014) and Fox and Bale (2017) suggest, for example, that sexuality refuses to be reduced to biology or culture and is instead made up of a complex matrix of relations in an event assemblage. These assemblages involve humans—family, peers, teachers, other social practices of alcohol, cars and condoms, moral standards, institutions (school, family, the media) and social norms and values as well as materials—such as technology (Fox & Bale, 2017, p. 405). These assemblages are in a constant process of assembling and dis‐assembling. This means the ‘capacities to do, think, feel emerge and recede according to the mix, at any one moment, at any given time or place.

The focus for research is then at the level of these actions or practices, interactions and events. It includes the consistent, fairly stable and yet ever emergent and changing flux and flow of affects, capacities and patterning. These are produced by sociocultural and biological norms and understandings. These affect economies and the micropolitics that accompany them suggest potentialities for both conservative and progressive change (Fox & Alldred, 2014). Even with these insights, assemblages can persist to narrowly define or constrain phenomena like sexuality, gender or intersex variance. Moreover, the notion of power and the importance of those relations or mechanisms in these assemblages is rarely considered fully or directly in these theories (Andrews & Duff, 2019). Instead they would be conceived as further sets of affects in specific assemblages that are producing capacities to feel, think and do and be. Utilising the concept of assemblages and materialist theories for those living with sex‐variant bodies would conceive their bodies and lives as always becoming, materialising dynamically, occupying locations and always constituted through historical, social and political economic processes and environments. The related attributes or properties of an intersex self or body assemblage are never discrete, but instead comprise circuits of material bodies, institutions, affect and relations that enfold, enmesh and intertwine (Malatino, 2019). Such a theoretical framing begins to build an alternative vocabulary to speak about queer or intersex selfhood. It would allow us to consider health equity more fully for those with intersex variance as forms of emplaced‐in‐place‐embodiments, identities and potentially avoid a denigrating alterity.

### A ‘choreography’ of matter and social change

New materialist thinking and the concept of assemblages also suggest a flat ontology. That is where we avoid a human superior alterity or centrality to our research whilst aiming to represent the assemblages relevant to sex‐variant people’s concerns. Malatino (2019, p. 210) argues that to fully understand sex variance, we need to avoid the ‘marginality centricity dyad’ or the metaphor of centre and margin. Nor should we reduce such experiences to simplistic accounts of vulnerability or as forms of victimhood. For people with intersex variance, they advocate we avoid too neat a divide between normative and resistance—a division they argue that is ‘ill‐equipped to consider the complex complicities and concessions all subjects are forced to make in late capitalist neoliberal milieus’ (Malatino, 2019, p. 210). Moreover, given that new materialist thinking suggests matter, including the body has agency, that active lively nature also has the potential to evade, hinder, even defy human intention. This is evident, for example, in encounters with technology or during the COVID‐19 pandemic and with climate change. This suggests a complex ‘choreography’ of matter, which has consequences for how change is conceived and understood (Coole & Frost, 2010, p. 9). Moreover, when arguing for new materialism as a frame for intersex health research, we also heed the caution that much posthuman theoretical endorsement focuses on the positive, but very little writing or research attends to the negative. The destructive controlling assemblages, their compositions and energies, ways in which institutional power still operates are rarely researched using these theories, or when pain, fear and stress exist and what this looks like in a more than human understanding (Andrews & Duff, 2019). Such theoretical framings would need to pay more detailed attention to the damaging effects of these specific assemblages.

Forming part of wider assemblages of relations, sex‐variant bodies are understood not in terms of what they are, but in terms of what they can do. This means the focus of inquiry would concern how sex variance is produced and materialised via social, cultural and material relations. The body would be conceived as being in a process of becoming via these sociomaterial entanglements and in creating health. With ontological privilege removed from human agency, we can recognise how non‐human matter influences the ability or capability of the human body (Fox & Powell, 2021). With materiality as agentive and entangled with meaning, the sex‐variant body emerges from intra‐actions with other health care‐related objects and subjects. This theoretical framing may offer more subtle readings of difference and may constitute engagement, which is more ethically accountable. This kind of research acts as a modest witness, whilst being accountable to those experiencing such damaging inequalities (Haraway, 2008).

### Evolving embodied legibility

Like Malatino (2019), using these theories means we are less focussed on sex variance as ontological and epistemological objects of study and more concerned with the theoretical, political and conceptual work that sex variance does. This thinking also suggests how such understandings have the potential to enable or disrupt normative practices that act as a barrier to equity in health care contexts. Moreover, new materialist explanations offer the potential to read intersex embodiment as exceeding notions of subjective legibility, gendered realness or sexed materiality by reworking what it means to have a legible body in the West. Traditionally, embodied legibility is contingent on binary sex in so far as being either born a woman or a man whilst continuing to present in that way (Barad, 2007; Malatino, 2019). Due to intersex embodiment disrupting notions of both binary sex and gender, with new materialism, we read sex variance as part of broader emerging socio‐material assemblages. The body remains unfinished or evolving through the enactment of gender or gender performativity and can destabilise binaries of sex (Malatino, 2019). This process is reflected in the activism of the gender variant visual artist Del LaGrace Volcano, who magnifies rather than erases the intersex fragments of their body. This kind of visual activism rejects the binary logic with the accompanying surgical and hormonal interventions. It distorts binary scripts for embodiment but without arriving at any stable or fixed sexed self. In doing so, the process of becoming represents a refusal to endorse any stable ontology. For Volcano, in some instances, passing as either male or female without being based on any essence due to being either or both, makes life liveable. Both forms of passing as male or as female, however, require a level of compromise in having to reluctantly conform to the normative (Malatino, 2019).

### Health equity and intersectionality

Inequalities in health and health care are known to be mutually constituted through intersections of difference and privilege that shape people’s lives, locations and experiences (Cho et al., 2013; Crenshaw, 1991; Davis, 2015). Intersectionality supports an understanding of the sociomaterial factors for both what is privileged, or who or what has agency (Crocetti et al., 2021). This includes who or what is marginal, such as gender and sex and may be subject to discrimination. The response to such markers of difference and the transformation required for health equity varies globally according to legal, political and economic factors. Equalities legislation, for example, can either protect people or prohibit people from full participation in mainstream life (Zeeman et al., 2019). Additionally, the well‐known sociomaterial determinants of poverty, living conditions, housing, access to employment, health care and education (Harris et al., 2020) are interwoven with the processes of how sociomaterial benefits and limitations are established. As the range of cultural and social perspectives on intersex variance expands (Monro et al., 2021), and acknowledges the importance of agency (Crocetti et al., 2021), the possibilities for health equity increase, especially when co‐produced with sex‐variant people. Yet future research must also take into account the above intersectional capacities and differences that are not just the outcomes of more modern notions of latent structures or forces. These accounts must instead expose the emergent and materialising nature of intersectional differences entangled with meaning and matter.

### Health equity and queer retheorising of the sex binary

Considerations of health equity allow for a shift in the way sex variance is framed whilst questioning the ethical accountability of unwanted medical and health care interventions. Critical intersex studies have shown the damaging effects of medical histories and how activist interventions, in diagnosis and treatment, challenge and more positively focus on capacities and strengths (Malatino, 2019). Exploring how subjects construct and make meaning of their lives and related health experiences reveals how these are shaped by the way medical and scientific epistemologies utilise knowledge and power relations to diagnose and understand intersex conditions. Scientific biomedical diagnosis frames intersex variance as ‘disorders of sex development (DSD), where intersex embodiment risks being ‘trapped’ in linguistic and discursive binaries. However, challenges to these binaries indicate how sex variance can be understood as destabilising notions of an assumed ‘normal’ body (Fausto‐Sterling, 2000). This impasse between medical pathologisation and social cultural theories attempts to overturn or trouble binaries has led to queering intersex embodiment (Malatino, 2019). In these theories, the aim is not only to disrupt or deconstruct entrenched binaries but also reframe dominant sex and gender discourse (Grimes, 2016). To replace such accounts and effects, Malatino (2019) asserts in ‘Queer embodiment: monstrosity, medical violence and intersex experience’ centring ontology by asking—*‘What is it to be intersex?*’ (ibid 2019, p. 1).

However, such attempts to remove or erase distinctions to resolve binary or dualistic thinking, some may argue, has never completely succeeded (Gunnarsson, 2013). As Maria Lugones (2010) suggests, central to modernity’s categorical, dichotomous, hierarchical logic is colonial capitalist thinking about gender and sexuality. This produces the tensions and resistance noted above. While Frost (2014) shows how, despite activist and academic attempts to open up the binary, most discussions of non‐normative gender or sexualities and bodies tend to retain the underpinning dualism of male‐female as the system of reference. Even with decades of scholarship seeking to critique, deconstruct and expose the ‘naturalness’ of the heterosexual dual‐sex system, it remains stubbornly in place. This is even so when theorised as socially, culturally and discursively constructed, or viewed as a truth claim and practice of power, or as empirically untrue. Trying to displace or remove the binary has proved challenging. As Zerilli (2005) and Frost (2014) show, the sex‐gender system provides a basic set of coordinates and background meanings to make sense of sex and gender, which in turn limits attempts to deconstruct and denaturalise these concepts. Theoretically, however, Frost (2014) proposes bodies have a fundamental permeability in that they live and die within, and are shaped by, a material world. This living body and its material and social context, entangled together, elicit and produce a continually changing identity, body and form (Frost, 2014, p. 319). In other words, as proposed by new materialism, biology and social processes are inseparable forces and should both be at the centre of health‐related research for intersex‐variant people.

In sum, at the centre of our argument is the need for new frames of reference, both theoretically and conceptually. This is needed to make sense of how material and sociocultural processes work together, how the social and material interact, as Butler argued (1993). Reformulation will produce understandings of bodies that matter, and in this paper, how sex‐variant embodiment with experiences of sex‐gender identities are embedded within and have entangled natureculture or biocultural dimensions. A convergence of material feminism, new materialism with the turn to biology or matter offers such a frame. We argue these theories suggest a more theoretically informed and detailed understanding of the potential of intersex people’s lived experiences and their bodies for what they can be, feel, think and do.

This more nuanced understanding of what sex variant bodies offer as part of broader bodies‐health‐equity‐assemblages is an attempt to move beyond the oft cited academic, medical and activist ‘either/or’ debates of celebration or problematisation. Nevertheless, there is caution over the use of ‘new’ in these theories. Most accounts cited here emanate from the global north and can end up offering white ethnocentric perspectives that further retain a lurking humanism whilst overlooking other knowledge and values arising from differing, relational understandings of the world, such as indigenous or spiritual epistemologies. Whilst humanism as an undercurrent in western research is appreciated by some, the theories we wish to draw on challenge the ontological limitations assumed by modern logocentric thought and a stable, rational, coherent and human subject (Andrews & Duff, 2019; Braidotti, 2013). Whilst the full implications of such thinking have yet to be realised, there remain important questions over whether such micro‐detailed explorations will serve to ignore broader, large‐scale patterns and trends of oppression and discrimination.

### A new research agenda

In arguing for further theorising and research, we hope to avoid a reductive framing of intersex people’s lives (Monro et al., 2021). In exploring recent theories of matter or materialism being revised in the ‘turn to matter’ (Barad, 2003), we combined this with the valuable insights of the cultural turn within critical intersex studies, with poststructural and queer theorising of discursive understandings of subjectivities, identities and embodiment. This provides a new agenda for co‐produced intersex research, which positively focuses on the concerns of people with intersex variance and what intersex bodies can be, know, or do in the spaces and places where they access health care. Conceiving of matter, including bodies and spaces, as more than a mere backdrop or outside of embodied experience, we acknowledge a new potential to understand health equity. As shown in this paper, intersex activists and academics have long fought to promote the rights to bodily autonomy, and put an end to harmful non‐consensual, irreversible medical interventions, including unwanted surgery early in life. Advances in the protection of the right to bodily autonomy that resist unnecessary surgery for children with intersex variance are reflected in this quote of a consultation between a doctor and the parents of a child who presented with intersex variance at birth.

I say what I always say, which is “we are happy that your child is healthy. We don’t need to do anything. Everything is fine. Look at your beautiful child”. I will say this several times, so it is clear that there is no deal [no rush to do anything] now. So they will not do anything, I tell them what I think is most important so they can connect to their child.

(Dr. E., 28 November 2015 in Meoded Danon, 2019, p. 155)

This interaction reflects the protection of bodily autonomy with appreciation for the birth of a child with the potential of life and connection where sex‐variant embodiment is understood and normalised. However, any future research focussing on intersex variance should recognise the fragility of legal protection for their rights to bodily autonomy that varies globally (FRA, 2012; ILGA‐Europe, 2015; Zeeman & Aranda, 2020). Recent legal challenges have had some success and have led to the creation of a third sex category in Germany and treatment as agentic subjects entitled to choice (von Wahl, 2019). Whilst these rights and legal recognition are enshrined in Australia, Germany and Malta (Garland & Travis, 2018), these advances may perpetuate notions of victimhood or an assumed vulnerability that should be resisted (Garland & Travis, 2018).

Paradoxically, the ongoing challenge to societal and medical pathologised understandings of what a sexed body is has resulted in more strength‐ or asset‐based approaches to understanding intersex variation. Importantly, this research questions or challenges normative understandings of embodiment (Lundberg et al., 2018; Meoded Danon, 2019). Given the many different embodied experiences of intersex variance (Fausto‐Sterling, 2000; Jones, 2018), we call for further parent or patient‐centred, community‐driven research to understand how people navigate their way through health settings, places, structures and systems. Research should consider when and where health care spaces may facilitate or possibly produce embodied wellbeing whilst explicitly seeking to develop co‐produced research that is meaningful and relevant to people with intersex variance. Emerging from this framing, we propose the following key areas for future research in consultation with and peer involvement of people with intersex variance:

- Through strength‐ or asset‐based research, exploring the capacities for non‐normative understandings of sex and gender.
- Examining intersex embodiment and subjectivity in health care as multi‐layered interdependence via socio‐material assemblages.
- Understanding the capacities for health equity stemming from the benefits and drawbacks during the interchange between human and non‐human matter.

## CONCLUSION

As we have argued, new materialist theorising will acknowledge matter and meaning as inseparable in the assemblages or spaces or places of health care. These theories suggest a new ethical way of living life, of witnessing and of being with the material and non‐material world. This view connects sex‐variant embodiment to histories of pain and collective histories of oppression, as well as to bodies that know and have capacities to be and do. Health‐related settings are always connected to sensory, biological bodies, intersected by differences. However, these histories also provide grounds to allow people to orient towards spaces that allow lives to flourish or reach for support to achieve such ends. Similarly, this theorising reveals how materiality may define people as different and problematic, but equally offers plural possibilities that remain open and indefinite too, as mutually constituted via body‐mind‐environment relations.

A new agenda of co‐produced research will focus on the concerns of people with intersex variance and what sex‐variant bodies can be, know, or do. Through capacity‐based research or via system‐based research, we can achieve greater understanding of the socio‐material assemblages of intersex materiality in health and health care from a position of strength rather than a presumption of vulnerability or victimhood. Theorising health equity research with new materialisms will aid understanding of the material and embodied realities of people with intersex variance. Research informed by new materialism should be undertaken with a specific focus on intersex health as relational entanglements mutually constituted between the material and social worlds. These forms of research should take place in consultation with, and peer involvement of, people with intersex variance as they understand their own health needs and experiences of accessing services. For health research, we propose to investigate how the related meanings or understandings of intersex subjectivity and embodiment materialise as multi‐layered forms of interdependence in assemblages. How these more complete assemblages of bodies, biomedical technologies, objects and spaces join to inform health. Finally, we argue for research to reconsider the sociomaterial aspects of health inequalities, with greater awareness of the agentic interchange and transformative capacities between matters and meanings in the advance towards health equity.

## AUTHOR CONTRIBUTIONS

**Laetitia Zeeman**: Conceptualization (Lead); Formal analysis (Equal); Investigation (Equal); Resources (Equal); Software (Equal); Visualization (Equal); Writing – original draft (Lead); Writing – review & editing (Equal). **Kay Aranda**: Conceptualization (Supporting); Formal analysis (Equal); Resources (Equal); Software (Equal); Supervision (Lead); Validation (Lead); Writing – original draft (Supporting); Writing – review & editing (Equal).

## Data Availability

As the paper is informed by theoretical and research literature the authors did not collect any primary research data.

## References

[shil13561-bib-0001] Alaimo, S. , & Hekman, S. (Eds.). (2008). Material feminisms. Indiana University Press.

[shil13561-bib-0002] Alldred, P. , & Fox, N. (2017). Young bodies, power and resistance: A new materialist perspective. Journal of Youth Studies, 20(9), 1161–1175. 10.1080/13676261.2017.1316362

[shil13561-bib-0003] Andrews, G. , & Duff, C. (2019). Matter beginning to matter: On posthumanist understandings of the vital emergence of health. Social Science & Medicine, 226, 123–134. 10.1016/j.socscimed.2019.02.045 30852392

[shil13561-bib-0004] Aranda, K. (2019). The political matters: Exploring material feminist theories for understanding the political in health, inequalities and nursing. Nursing Philosophy, 20(4). 10.1111/nup.12278 31364816

[shil13561-bib-0005] Barad, K. (1998). Getting real: Technoscientifc practices and the materialization of reality. A Journal of Feminist Cultural Studies, 10(2), 87–128. 10.1215/10407391-10-2-87

[shil13561-bib-0006] Barad, K. (2003). Posthumanist performativity: Toward an understanding of how matter comes to matter. Signs: Journal of Women in Culture and Society, 28(3), 801–831. 10.1086/345321

[shil13561-bib-0007] Barad, K. (2007). Meeting the universe halfway: Quantum physics and the entanglement of matter and meaning. Duke University Press.

[shil13561-bib-0008] Barad, K. (2012). Matter feels, converses, suffers, desires, yearns and remembers. In R. Dolphijn & I. van der Tuin (Eds.), New materialisms: Interviews and cartographies (pp. 48–70). Open Humanities Press.

[shil13561-bib-0009] Bendelow, G. (2009). Health, emotion and the body. Polity.

[shil13561-bib-0010] Braidotti, R. (2013). The posthuman. Polity.

[shil13561-bib-0011] Braidotti, R. (2019). A theoretical framework for the critical posthumanities. Theory, Culture & Society, 36(6), 31–61. 10.1177/0263276418771486

[shil13561-bib-0012] Buchanan, I. (1997). The problem of the body in Deleuze and Guattari, or, what can a body do? Body & Society, 3(3), 73–91. 10.1177/1357034x97003003004

[shil13561-bib-0013] Butler, J. (1993). Bodies that matter: On the discursive limits of ‘sex’. Routledge.

[shil13561-bib-0014] Butler, J. (2004). Undoing gender. Routledge. ISBN: 978‐0‐203‐49962‐7.

[shil13561-bib-0015] Cho, S. , Crenshaw, K. , & McCall Source, L. (2013). Toward a field of intersectionality studies: Theory, applications, and praxis. Signs, 38(4), 785–810. 10.1086/669608

[shil13561-bib-0016] Coole, D. , & Frost, S. (Eds.). (2010). New materialisms: Ontology, agency, and politics. Duke University Press.

[shil13561-bib-0017] Crenshaw, K. (1991). Mapping the margins: Intersectionality, identity politics, and violence against women of color. Stanford Law Review, 43(6), 1241–1299. 10.2307/1229039

[shil13561-bib-0018] Crocetti, D. , Monro, S. , Vecchietti, V. , & Yeadon‐Lee, T. (2021). Towards an agency‐based model of intersex, variations of sex characteristics (VSC) and DSD/dsd health. Culture, Health and Sexuality, 23(4), 500–515. 10.1080/13691058.2020.1825815 33236685

[shil13561-bib-0019] Davis, K. (2015). Who owns intersectionality? Some reflections on feminist debates on how theories travel. European Journal of Women's Studies, 27(2), 113–127. 10.1177/1350506819892659

[shil13561-bib-0020] Deleuze, G. (1988). Spinoza: Practical philosophy. City Lights Publishers.

[shil13561-bib-0021] Fausto‐Sterling, A. (2000). Sexing the body: Gender politics and the construction of sexuality. Basic Books.

[shil13561-bib-0022] Fox, N. (2016). Health sociology from post‐structuralism to the new materialisms. Health, 20(1), 62–74. 10.1177/1363459315615393 26572797

[shil13561-bib-0023] Fox, N. (2017). Personal health technologies, micropolitics and resistance: A new materialist analysis. Health, 21(2), 153. 10.1177/1363459315590248 26216896

[shil13561-bib-0066] Fox, N. J. , & Alldred, P. (2014). New materialist social inquiry: designs, methods and the research‐assemblage. International Journal of Social Research Methodology. 10.1080/13645579.2014.921458

[shil13561-bib-0067] Fox, N. J. , & Alldred, P. (2016). Sociology, environment and health: A materialist approach. Public Health, 141, 287–293. 10.1016/j.puhe.2016.09.015 27780587

[shil13561-bib-0026] Fox, N. J. , & Bale, C. (2017). Bodies, pornography and the circumscription of sexuality: A new materialist study of young people’s sexual practices. Sexualities, 21(3), 393–409. 10.1177/1363460717699769

[shil13561-bib-0024] Fox, N. , & Alldred, P. (2013). The sexuality‐assemblage: Desire, affect, anti‐humanism. Sociology Review, 61(4), 769–789. 10.1111/1467-954x.12075

[shil13561-bib-0025] Fox, N. , & Powell, K. (2021). Non‐human matter, health disparities and a thousand tiny dis/advantages. Sociology of Health & Illness, 43(3), 1–17. 10.1111/1467-9566.13265 33720407

[shil13561-bib-0027] FRA . (2012). Handbook on European non‐discrimination law: Case‐law update. European Union Agency for Fundamental Rights.

[shil13561-bib-0028] Frost, S. (2014). Re‐considering the turn to biology in feminist theory. Feminist Theory, 15(3), 307–326. 10.1177/1464700114545323

[shil13561-bib-0029] Fullagar, S. (2017). Post‐qualitative inquiry and the new materialist turn: Implications for sport, health and physical culture research. Qualitative Research in Sport, Exercise and Health, 9(2), 247–257. 10.1080/2159676x.2016.1273896

[shil13561-bib-0030] Garland, F. , & Travis, M. (2018). Legislating intersex equality: Building the resilience of intersex people through law. Legal Studies, 38(4), 587–606. 10.1017/lst.2018.17

[shil13561-bib-0031] Gibson, B. E. , King, G. , Teachman, G. , Mistry, B. , & Hamdani, Y. (2017). Assembling activity/setting participation with disabled young people. Sociology of Health & Illness, 39(4), 497–512. 10.1111/1467-9566.1249 27868201 PMC5434907

[shil13561-bib-0032] Grimes, K. (2016). Theology of whose body? Sexual complementarity, intersex conditions, and La Virgen de Guadalupe. Journal of Feminist Studies in Religion, 32(1), 75–93. 10.2979/jfemistudreli.32.1.06

[shil13561-bib-0033] Gunnarsson, L. (2013). The naturalistic turn in feminist theory: A Marxist‐realist contribution. Feminist Theory, 14(1), 3–19. 10.1177/1464700112468567

[shil13561-bib-0034] Haraway, J. D. (2008). When species meet. University of Minnesota Press.

[shil13561-bib-0035] Harris, P. , Baum, F. , Friel, S. , MacKean, T. , Schram, A. , & Townsend, B. (2020). A glossary of theories for understanding power and policy for health equity. Journal of Epidemiology & Community Health, 74(6), 548–552. 10.1136/jech-2019-213692 32198290

[shil13561-bib-0036] Hassan, N. R. , Swartz, L. , Kagee, A. , De Wet, A. , Lesch, A. , Kafaar, Z. , & Newman, P. (2018). There is not a safe space where they can find themselves to be free: (Un)safe spaces and the promotion of queer visibilities among township males who have sex with males (MSM) in Cape Town, South Africa. Health & Place, 49, 93–100. 10.1016/j.healthplace.2017.11.010 29227887

[shil13561-bib-0037] ILGA‐Europe . (2015). Annual review of the human rights situation of lesbian, gay bisexual trans and intersex people in Europe. ILGA.

[shil13561-bib-0038] Jagger, G. (2015). The new materialism and sexual difference. Signs. Journal of Women in Culture and Society, 40(2), 321–341. 10.1086/678190

[shil13561-bib-0039] Jones, T. (2016). The needs of students with intersex variations. Sex Education, 16(6), 602–618. 10.1080/14681811.2016.1149808

[shil13561-bib-0040] Jones, T. (2018). Intersex: A systematic review of international health literature. Sage Open(8), 2158244017745577. 10.1177/2158244017745577

[shil13561-bib-0041] Köhler, B. , Kleinemeier, E. , Lux, A. , Hiort, O. , Gruters, A. , Thyen, U. , Akkurt, I. , Albers, N. , Bartsch, O. , Becker, D. , Beckmann, M. W. , Beetz, R. H. , Bettendorf, M. , Beyer, P. , Binder, G. , Boemers, T. , Brack, C. , Caliebe, A. , Dietz, H. G. , …, Schober, M. (2012). Satisfaction with genital surgery and sexual life of adults with XY disorders of sex development: Results from the German clinical evaluation study. The Journal of Clinical Endocrinology, (97), 577–588. 10.1210/jc.2011-1441 22090272

[shil13561-bib-0042] Lugones, M. (2010). Toward a decolonial feminism. Hypatia, 25(4), 742–759. 10.1111/j.1527-2001.2010.01137.x

[shil13561-bib-0043] Lundberg, T. , Hegarty, P. , & Roen, K. (2018). Making sense of ‘intersex’ and ‘DSD’: How laypeople understand and use terminology. Psychology & Sexuality, 9(2), 161–173. 10.1080/19419899.2018.1453862

[shil13561-bib-0044] Lupton, D. (2019). Toward a more‐than‐human analysis of digital health: Inspirations from feminist new materialism. Qualitative Health Research, 29(14), 1–12. 10.1177/1049732319833368 30964392

[shil13561-bib-0045] Malatino, H. (2019). Queer embodiment: Monstrosity, medical violence, and intersex experience. University of Nebraska Press.

[shil13561-bib-0046] Maller, C. , & Stengers, Y. (Eds.). (2019). Social practices and dynamic non‐humans: Nature, materials and technologies. Palgrave Macmillan.

[shil13561-bib-0047] Markula, P. (2019). What is new about new materialism for sport sociology? Reflections on body, movement, and culture. Sociology of Sport Journal, 36, 1–11. 10.1123/ssj.2018-0064

[shil13561-bib-0048] Marmot, M. , & Allen, J. (2014). Social determinants of health equity. American Journal of Public Health, 104 (S4), S517–S519. 10.2105/AJPH.2014.302200 25100411 PMC4151898

[shil13561-bib-0049] Meer, T. , & Muller, A. (2017). They treat us like we’re not there: Queer bodies and the social production of healthcare spaces. Health & Place, 45, 92–98. 10.1016/j.healthplace.2017.03.010 28324795

[shil13561-bib-0050] Meoded Danon, L. (2019). Comparing contemporary medical treatment practices aimed at intersex/DSD bodies in Israel and Germany. Sociology of Health & Illness, 41(1), 143–164. 10.1111/1467-9566.12812 30182487

[shil13561-bib-0051] Monro, S. , Carpenter, M. , Crocetti, D. , Davis, G. , Garland, F. , Griffiths, D. , Hegarty, P. , Travis, M. , Cabral Grinspan, M. , & Aggleton, P. (2021). Intersex: Cultural and social perspectives. Culture, Health and Sexuality, 23(4), 431–440. 10.1080/13691058.2021.1899529 33783329

[shil13561-bib-0052] Piketty, T. (2014). Capital in the twenty first century. President and Fellows of Harvard College.

[shil13561-bib-0053] Rosenwohl‐Mack, A. , Tamar‐Mattis, S. , Baratz, A. B. , Dalke, K. B. , Ittelson, A. , Zieselman, K. , & Flatt, J. D. (2020). A national study on the physical and mental health of intersex adults in the U.S. PLoS One, 15(10), e0240088. 10.1371/journal.pone.0240088 33035248 PMC7546494

[shil13561-bib-0054] Schweizer, K. , Brunner, F. , Handford, C. , & Richter‐Appelt, H. (2014). Gender experience and satisfaction with gender allocation in adults with diverse intersex conditions. Psychology and Sexuality, 5(1), 56–82. 10.1080/19419899.2013.831216

[shil13561-bib-0055] Thyen, U. , Lux, A. , Jürgensen, M. , Hiort, O. , & Köhler, B. (2014). Utilization of health care services and satisfaction with care in adults affected by disorders of sex development (DSD). Journal of General Internal Medicine, 29(S3), 752–759. 10.1007/s11606-014-2917-7 PMC4124114 25029980

[shil13561-bib-0056] von Wahl, A. (2019). From object to subject: Intersex activism and the rise and fall of the gender binary in Germany. Social Politics: International Studies in Gender, State & Society, 28(3), 1–23. 10.1093/sp/jxz044

[shil13561-bib-0057] Whitehead, M. (1991). The concepts and principles of equity and health. Health Promotion International, 6(3), 217–228. 10.1093/heapro/6.3.217 1644507

[shil13561-bib-0058] WHO . (2021). Health equity and its determinants. It’s time to build a fairer, healthier world for everyone, everywhere. World Health Organization. Available from www.who.int/health‐topics/health‐equity%20accessed%2027.07.22

[shil13561-bib-0059] Wilkinson, R. , & Pickett, K. (2018). Inner level: How more equal societies reduce stress, restore sanity and improve everyone’s wellbeing. Allen Lane. 10.3399/bjgp19X705377 PMC6715479 31467020

[shil13561-bib-0060] Zeeman, L. , & Aranda, K. (2020). A systematic review of the health and healthcare inequalities for people with intersex variance. International Journal of Environmental Research and Public Health, 17(18), 1–18. 10.3390/ijerph17186533 PMC7559554 32911732

[shil13561-bib-0061] Zeeman, L. , Aranda, K. , & Grant, A. (Eds.). (2014). Queering health: Critical challenges to normative health and healthcare. PCCS Books. ISBN: 978‐1‐90625407101.

[shil13561-bib-0062] Zeeman, L. , Sherriff, N. , Browne, K. , McGlynn, N. , Mirandola, M. , Gios, L. , Davis, R. , Sanchez‐Lambert, J. , Aujean, S. , Pinto, N. , Farinella, F. , Donisi, V. , Niedzwiedzka‐Stadnik, M. , Rosinska, M. , Pierson, A. , Amaddeo, F. , Taibjee, R. , Toskin, I. , Jonas, K. , …, De Sutter, P. (2019). A review of lesbian, gay, bisexual, trans and intersex (LGBTI) health and healthcare inequalities. The European Journal of Public Health, 29(5), 974–980. 10.1093/eurpub/cky226 30380045 PMC6761838

[shil13561-bib-0063] Zeiler, K. (2020). Why feminist technoscience and feminist phenomenology should engage with each other: On subjection/subjectivity. Feminist Theory, 21(3), 367–390. 10.1177/1464700120920763

[shil13561-bib-0064] Zerilli, L. (2005). Feminism and the abyss of freedom. Chicago University Press.

